# Synergistic Effects of Low-Frequency Ultrasound and Therapeutic Agents on Endothelial and Renal Cells: Emphasis on Cell Functionality, Oxidative Stress, and Inflammatory Markers

**DOI:** 10.3390/ph18030404

**Published:** 2025-03-13

**Authors:** Ieva Čiapienė, Joris Vėžys, Vaiva Lesauskaitė, Indrė Matulevičiūtė, Ugnė Meškauskaitė, Vilius Skipskis, Arvydas Strazdauskas, Sonata Trumbeckaitė, Algimantas Bubulis, Vytautas Jūrėnas, Vytautas Ostaševičius, Vytenis Tamakauskas, Vacis Tatarūnas

**Affiliations:** 1 Institute of Cardiology, Lithuanian University of Health Sciences, Sukileliu 15, LT-50103 Kaunas, Lithuania; ieva.ciapiene@lsmu.lt (I.Č.); vaiva.lesauskaite@lsmu.lt (V.L.); ugne.meskauskaite@lsmu.lt (U.M.); vilius.skipskis@lsmu.lt (V.S.); vytenis.tamakauskas@lsmu.lt (V.T.); 2 Department of Mechanical Engineering, Faculty of Mechanical Engineering and Design, Kaunas University of Technology, Studentu 56, LT-51424 Kaunas, Lithuania; joris.vezys@ktu.lt; 3 Department of Ophthalmology, Lithuanian University of Health Sciences, Eiveniu 2, LT-50161 Kaunas, Lithuania; indre.matuleviciute@lsmu.lt; 4 Department of Biochemistry, Faculty of Medicine, Lithuanian University of Health Sciences, Eiveniu 4, LT-50161 Kaunas, Lithuania; arvydas.strazdauskas@lsmu.lt; 5 Department of Pharmacognosy, Faculty of Pharmacy, Lithuanian University of Health Sciences, Sukileliu 13, LT-50162 Kaunas, Lithuania; sonata.trumbeckaite@lsmu.lt; 6 Institute of Mechatronics, Kaunas University of Technology, Studentu 56, LT-51424 Kaunas, Lithuania; algimantas.bubulis@ktu.lt (A.B.); vytautas.jurenas@ktu.lt (V.J.); vytautas.ostasevicius@ktu.lt (V.O.)

**Keywords:** low-frequency ultrasound, RAS inhibitors, captopril, losartan, dexamethasone, inflammation, HUVEC, RPTEC/TERT1

## Abstract

**Background:** Ischemic heart disease remains the leading cause of death worldwide, with coronary microvascular dysfunction (CMD) as a key complication after ST-elevation myocardial infarction (STEMI). Endothelial dysfunction contributes to CMD, impairing vascular tone and increasing inflammation. While angiotensin-converting enzyme (ACE) inhibitors and angiotensin II receptor blockers (ARBs) aid vascular health, their efficacy may improve with therapeutic ultrasound, which enhances drug delivery and endothelial response. This study explores the combined effects of ultrasound and pharmacological treatment on the ACE axis and inflammation in endothelial and renal cells. **Methods:** Human umbilical vein endothelial cells (HUVECs) and human renal proximal tubular epithelial cell line RPTEC/TERT1 were treated with captopril, losartan, and dexamethasone, alone or combined with low-frequency ultrasound (LFU). Cell viability and wound-healing assays assessed cellular function, while nitric oxide (NO) and reactive oxygen species (ROS) assays were used to evaluate redox signaling. Gene expression related to the ACE axis, inflammation, and vascular and renal cell function was analyzed via qPCR. **Results:** Captopril and losartan combined with LFU improved endothelial cell viability, wound healing, and NO production at various concentrations, whereas only losartan with LFU enhanced cell viability and wound healing in renal cells. Dexamethasone with LFU increased ROS levels and had variable effects on RPTEC/TERT1 cell survival. Gene expression analysis showed that LFU alone reduced pro-inflammatory markers *VCAM-1*, *ICAM-1*, and *PTGS2* in captopril-treated HUVECs and similarly affected *CYP4F2* in losartan-treated HUVECs. LFU also decreased *PTGS2* expression at higher dexamethasone concentrations. In RPTEC/TERT1 cells, LFU alone did not impact *SGLT2* or *GGT1* expression, but captopril with LFU downregulated *GGT1*, and dexamethasone with LFU upregulated *SGLT2* at higher concentrations. **Conclusions:** This study demonstrates that LFU enhances the effects of RAS inhibitors by promoting NO synthesis and reducing oxidative stress, while its combination with dexamethasone may have variable, potentially cytotoxic effects on renal cells. Gene expression patterns suggest LFU’s anti-inflammatory potential and its role in modulating drug efficacy.

## 1. Introduction

Ischemic heart disease constitutes the most significant burden of disease and is the leading cause of death globally [1], especially in low-income countries [2]. Myocardial ischemia has traditionally been linked to obstructive or non-obstructive coronary artery atherosclerosis [3]. With advancements in interventional methods, particularly percutaneous coronary intervention (PCI) for vascular reperfusion, mortality rates from cardiovascular disease have plateaued but are no longer declining as anticipated [4]. Coronary microvascular dysfunction (CMD) is a frequent complication following ST-elevation myocardial infarction (STEMI). Approximately one in four patients develop CMD after experiencing STEMI. In the first 12 months after PCI, patients with CMD face an increased risk of adverse cardiac events, which can increase their mortality [5].

Dysfunction of macrovascular and microvascular systems is a significant contributor to cardiovascular disease and is closely linked to the functionality of vascular endothelial cells [6,7]. Endothelial cells form a continuous monolayer that lines the luminal surface of blood vessels and are pivotal in modulating vascular tone and blood flow. These cells are integral to maintaining vascular homeostasis via multiple mechanisms, acting as both a mechanical and biological barrier between the vascular lumen and the underlying tissues. This barrier facilitates the dynamic exchange of molecules between the interstitial fluid and blood, particularly in the capillary beds, where their selective permeability and regulatory functions are essential for tissue homeostasis and systemic health [7,8,9].

Endothelial cells exhibit distinct phenotypic characteristics influenced by their specific vascular location [10]. In macrovascular dysfunction, endothelial cells often lose their capacity to synthesize nitric oxide (NO), a critical mediator for vasodilation and the regulation of inflammatory processes [11]. Consequently, vessels with dysfunctional endothelium become more susceptible to developing atherosclerotic lesions, which significantly increases the risk of coronary artery disease [12]. Impairment in microvascular function is associated with dysfunction in small blood vessels, including arterioles and capillaries. Hypertension, inflammatory processes, oxidative stress, and hyperglycemia reduce NO bioavailability and increase oxidative stress, disrupting capillary function and compromising tissue perfusion. CMD observed after PCI is linked to the development of atrial fibrillation, decreased left ventricular ejection fraction, and impaired circulation [13]. Impaired circulation amplifies localized tissue damage, hypoxia, and inflammatory responses, significantly contributing to the pathophysiology of heart failure and chronic kidney disease [14,15]. Research indicates a connection between impaired kidney function and microcirculatory diseases [16]. Chronic kidney disease was shown to decrease endothelium-related vasodilation and increase adhesion molecule expression and coagulation [17].

Pharmacological strategies aimed at modulating vascular function and managing hypertension play a crucial role in maintaining and potentially restoring vascular integrity and mitigating cardiovascular events. The underlying signaling pathways involved are intricate and multifaceted. Key regulatory mechanisms pertinent to vascular function and hypertension include the calcium signaling pathway, the NO–NO-sensitive guanylate cyclase (NOsGC)–cyclic guanosine monophosphate (cGMP) pathway, and the processes associated with vascular remodeling. The latter is typically irreversible [18]. In addition to these pathways, inflammation plays a pivotal role in vascular dysfunction and hypertension. Chronic low-grade inflammation contributes to endothelial dysfunction, arterial stiffness, and vascular remodeling [19]. The renin–angiotensin system (RAS) is pivotal in mediating these effects. Angiotensin II (Ang II), a key effector of RAS, promotes vascular inflammation by activating pro-inflammatory pathways, including the nuclear factor-kappa B (NF-κB) signaling cascade. This activation leads to an increased expression of cytokines and adhesion molecules, exacerbating endothelial dysfunction [20]. Ang II also stimulates prostaglandin-endoperoxide synthases, increasing the synthesis of pro-inflammatory prostaglandins [21]. Angiotensin-converting enzyme (ACE) inhibitors and Ang II receptor blockers (ARBs) play a crucial role in reestablishing and sustaining the functional integrity of the vascular endothelium. Their mechanisms of action are to lower blood pressure and promote endothelial health, thereby enhancing NO availability and improving vascular reactivity. ACE inhibitors suppress Ang II formation and enhance bradykinin activity. ARBs block Ang II receptors AT_1_R and stimulate AT_2_R and AT_4_R, but the effect of ARBs was shown to be less potent. Along with affecting vasculature, ACE inhibitors and ARBs have been shown to preserve renal function and have protective effects in chronic kidney disease [22,23]. Various other compounds can influence vascular and renal health, including antioxidants, metabolic modulators, and anti-inflammatory agents like the corticosteroid dexamethasone, which exerts complementary effects on inflammation, oxidative stress, and the RAS [24]. Due to the variability in patient-specific characteristics, pharmacological agents exhibit differential effects across individuals. Several factors, including genetic makeup, metabolic pathways, and comorbid conditions, can influence the pharmacodynamic and pharmacokinetic responses to these drugs [25].

Non-invasive methods are gaining significant attention due to their potential to improve therapeutic outcomes, offering a safer and more patient-centered alternative. A promising approach to enhancing the therapeutic effects of ACE inhibitors and ARBs on endothelial and renal cell function may lie in combining these drugs with therapeutic ultrasound. When combined with systemically administered drugs, its synergistic potential has been extensively studied. This strategy facilitates the targeted activation of the vascular endothelium, thereby enhancing drug delivery while reducing off-target effects. Ultrasound may enhance endothelial function by increasing NO production, improving vascular tone, and reducing oxidative stress, which are key factors in endothelial dysfunction and CMD [26]. Previous studies have demonstrated ultrasound to have anti-inflammatory properties by downregulating adhesion molecule expression and reducing reactive oxygen species (ROS) accumulation in cell cultures [27,28]. In renal cells, ultrasound can influence mechanotransduction pathways, potentially modulating sodium and water reabsorption, as well as inflammatory responses [29]. For ultrasound to be applied safely and effectively, it is essential to accurately characterize energy transmission in biological tissues, striking a balance between electrical and acoustic impedance for optimal transduction efficiency. Ultrasound influences vascular contraction and relaxation through thermal, chemical, and mechanical mechanisms. While the thermal effects of ultrasound have been well researched, there is growing interest in its non-thermal effects, particularly concerning drug delivery [30].

Since the kidney is central to regulating RAS and is a target for RAS inhibitors, studying endothelial and renal cells together may provide a more comprehensive understanding of vascular health and therapeutic interventions with ultrasound. Thus, given the potential benefits and concerns raised, this study aimed to evaluate the synergistic effects of low-frequency ultrasound (LFU) and pharmacological treatment of RAS inhibitors and dexamethasone on endothelial and renal cell function, with a focus on viability, wound healing, redox signaling, ACE axis, and inflammation.

## 2. Results

### 2.1. Assessment of Cell Function Following Treatment with Captopril, Losartan, and Dexamethasone, Combined with LFU

Cell viability, wound healing, NO, and ROS production were assessed to evaluate endothelial and renal cell function after treatment with therapeutic compounds combined with LFU. The LFU treatment alone did not impact human umbilical vein endothelial cell (HUVEC) or RPTEC/TERT1 cell viability (Figures S1 and S2). However, captopril and LFU significantly increased HUVEC viability with all concentrations compared to the LFU-only control (Figure S1A). At the same time, losartan enhanced metabolic viability in a dose-dependent manner (Figure S1B). In RPTEC/TERT1 cells, losartan with LFU increased viability with all tested concentrations (Figure S2B), whereas higher dexamethasone doses (1 μM and 10 μM) with LFU reduced viability (Figure S2C).

Wound closure was documented 4 h after wounding initiation with each tested condition. Captopril (1.25–2.25 μM) with LFU enhanced HUVECs’ wound healing compared to the LFU-only control (Figure S3A). Higher concentrations of losartan also improved wound healing, with significant differences observed at 2.25–2.75 μM depending on LFU use (Figure S3B). Conversely, in RPTEC/TERT1 cells, only losartan (1.75–2.75 μM) with LFU significantly enhanced wound healing, with 2.25 μM showing the highest effect (Figure S4B).

Redox signaling molecules, NO for HUVECs and ROS for RPTEC/TERT1 cells, were assessed in response to treatment with the compounds and LFU. Captopril with LFU increased NO production in HUVECs dose-dependently, with significant effects at 1.75 and 2.25 μM (Figure S5A). Losartan with LFU enhanced NO levels at 2.25 and 2.75 μM, while LFU alone had no impact (Figure S5B). ROS production in RPTEC/TERT1 cells was measured after 1 h of treatment with the compounds, with or without LFU, followed by incubation with a ROS detection mix. Captopril with LFU significantly reduced ROS levels at 1.75 and 2.25 μM (Figure S6A). Losartan with LFU also decreased ROS production at higher concentrations (1.75–2.75 μM), while LFU alone had no impact (Figure S6B). Conversely, dexamethasone (0.1–10 μM) with LFU increased ROS production, with significant differences observed at lower concentrations (0.001 and 0.01 μM) (Figure S6C).

### 2.2. Effects of Captopril, Losartan, and Dexamethasone Combined with LFU on the mRNA Expression of Genes Related to Inflammation in HUVECs

**Figure 1:**
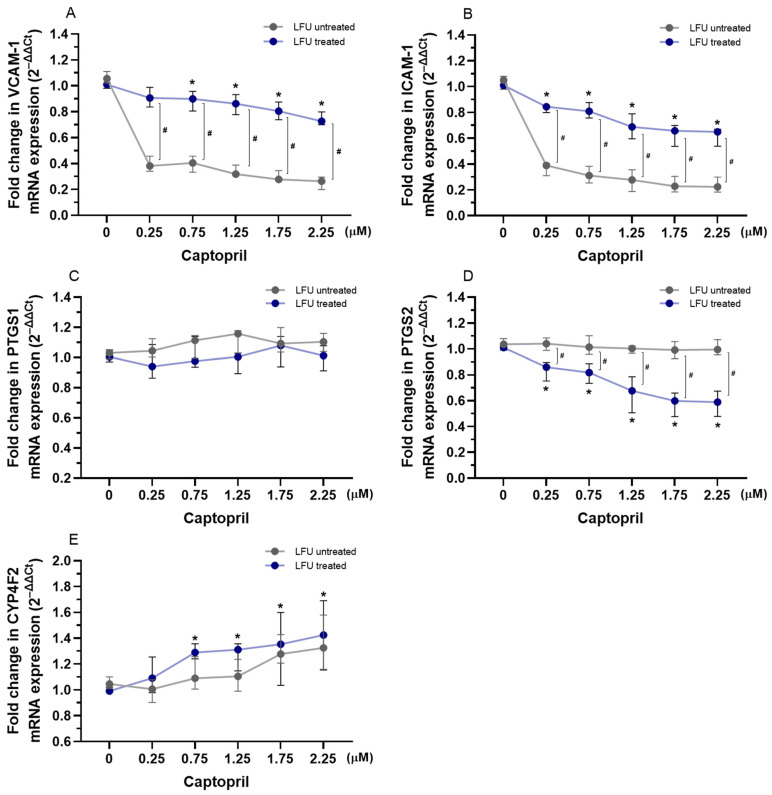
(**A**–**E**) Changes in the expression of *VCAM-1*, *ICAM-1*, *PTGS1*, *PTGS2*, and *CYP4F2* genes in HUVECs treated with therapeutic concentrations of captopril (0.25–2.25 μM) in combination with LFU. Gene expression changes are presented as fold changes compared to captopril untreated control cells (0 μM) exposed to LFU. Gene expression is normalized to the endogenous control gene *TFRC.* Data are expressed as median with range (*n* = 4). Values lower than *p* < 0.05 are indicated by (*) for comparisons within LFU-treated groups (different concentrations vs. LFU-only control) and by (#) for comparisons between LFU-treated and LFU-untreated groups.

To evaluate the impact of captopril, losartan, and dexamethasone combined with LFU on the transcription of *VCAM-1*, *ICAM-1*, *PTGS1*, *PTGS2*, and *CYP4F2* genes, the mRNA expression levels in HUVECs were analyzed. As shown in Figure 1, using the LFU influenced the expression of *VCAM-1*, *ICAM-1*, and *PTGS2* in HUVECs. Treatment with captopril and LFU reduced the expression of *VCAM-1*, *ICAM-1*, and *PTGS2* in a dose-dependent manner compared to control cells only treated with LFU (Figure 1A,B,D). In comparison, captopril and LFU did not affect *PTGS1* mRNA levels (Figure 1C). Treatment with (0.75–2.25 μM) captopril and LFU was observed to increase *CYP4F2* expression compared to the control group (Figure 1E).

**Figure 2:**
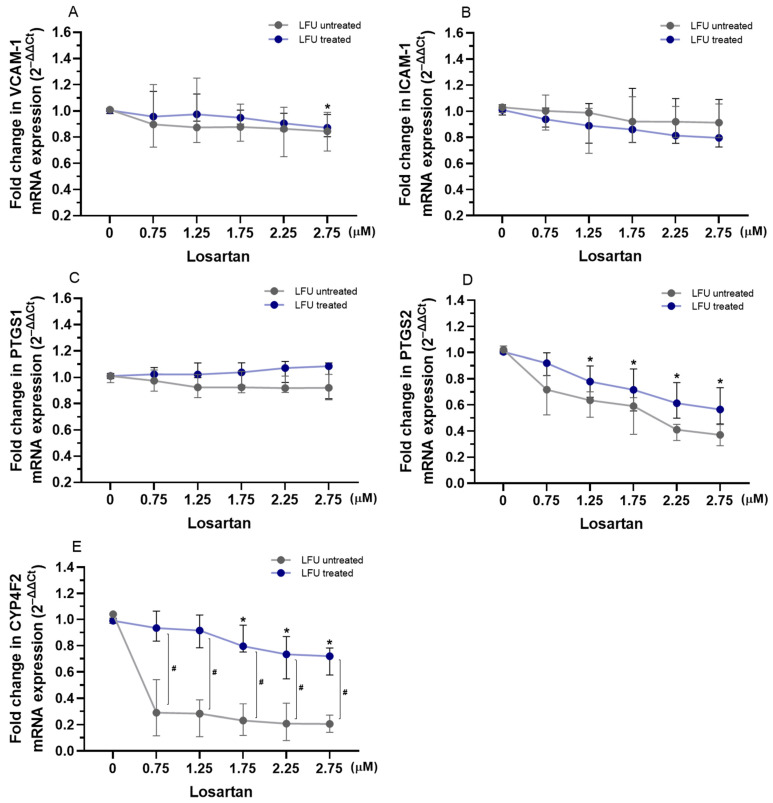
(**A**–**E**) Changes in the expression of *VCAM-1*, *ICAM-1*, *PTGS1*, *PTGS2*, and *CYP4F2* genes in HUVECs treated with therapeutic concentrations of losartan (0.75–2.75 μM) in combination with LFU. Gene expression changes are presented as fold changes compared to losartan untreated control cells (0 μM) exposed to LFU. Gene expression is normalized to the endogenous control gene *TFRC.* Data are expressed as median with range (*n* = 4). Values lower than *p* < 0.05 are indicated by (*) for comparisons within LFU-treated groups (different concentrations vs. LFU-only control) and by (#) for comparisons between LFU-treated and LFU-untreated groups.

Losartan combined with LFU did not impact *ICAM-1* or *PTGS1* mRNA levels in HUVECs (Figure 2B,C). Only treatment with the highest losartan concentration (2.75 μM) reduced the expression of *VCAM-1* compared to a control group treated with LFU (Figure 2A). Moreover, treatment with losartan and LFU reduced the expression of *PTGS2* in a dose-dependent manner compared to the control group (Figure 2D). Treatment with losartan showed significant differences in *CYP4F2* mRNA levels depending on the usage of LFU. However, only 1.75–2.75 μM concentrations of losartan reduced the expression of *CYP4F2* compared to the control group (Figure 2E).

**Figure 3:**
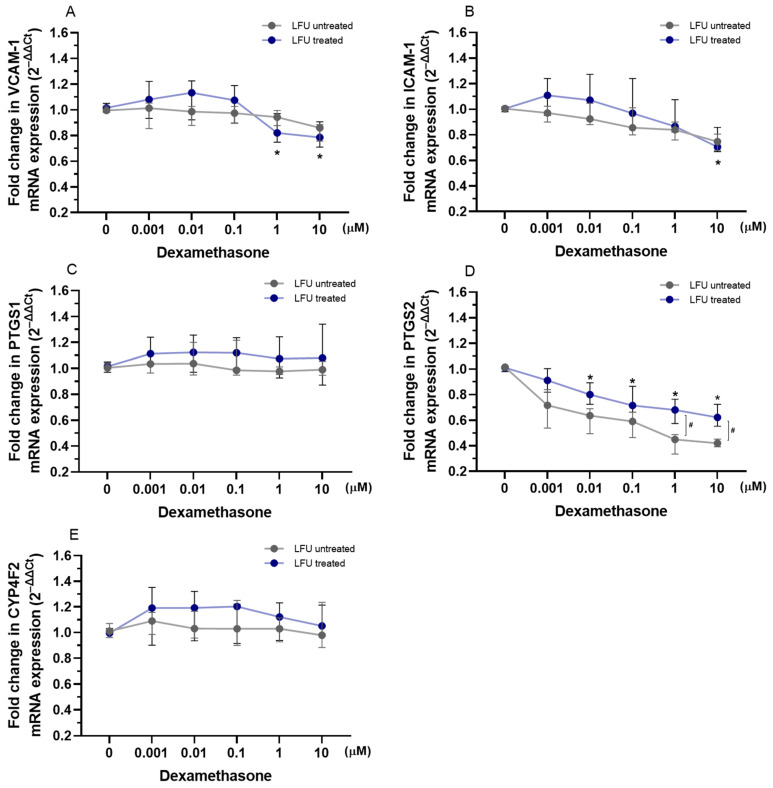
(**A**–**E**) Changes in the expression of *VCAM-1*, *ICAM-1*, *PTGS1*, *PTGS2*, and *CYP4F2* genes in HUVECs treated with dexamethasone (0.001–10 μM) in combination with LFU. Gene expression changes are presented as fold changes compared to losartan untreated control cells (0 μM) exposed to LFU. Gene expression is normalized to the endogenous control gene *TFRC.* Data are expressed as median with range (*n* = 4). Values lower than *p* < 0.05 are indicated by (*) for comparisons within LFU-treated groups (different concentrations vs. LFU-only control) and by (#) for comparisons between LFU-treated and LFU-untreated groups.

Dexamethasone combined with LFU reduced the expression of *VCAM-1* with 1–10 μM concentrations used, while the expression of *ICAM-1* was downregulated only with 10 μM of dexamethasone compared to control cells exposed to LFU (Figure 3A,B). In contrast, treatment with dexamethasone and LFU had no impact on *PTGS1* and *CYP4F2* mRNA levels (Figure 3C,E). The combination of 0.01–10 μM dexamethasone with LFU downregulated the expression of *PTGS2* (Figure 3D). Only treatment with higher concentrations of dexamethasone showed significant differences depending on the usage of LFU.

### 2.3. Effects of Captopril, Losartan, and Dexamethasone Combined with LFU on the mRNA Expression of Genes Related to Kidney Proximal Tubule Function in RPTEC/TERT1 Cells

**Figure 4:**
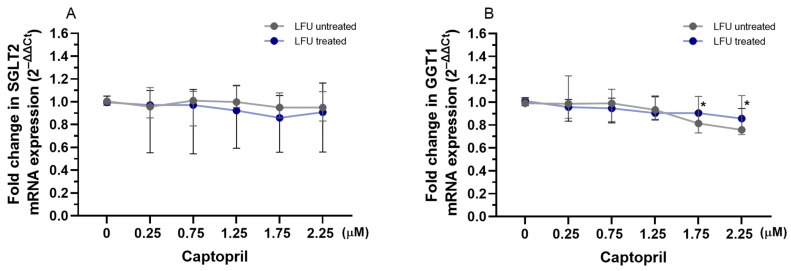
(**A**,**B**) Changes in the expression of *SGLT2*, *GGT1* genes in RPTEC/TERT1 treated with therapeutic concentrations of captopril (0.25–2.25 μM) combined with LFU. Gene expression changes are presented as fold changes compared to captopril untreated control cells (0 μM) exposed to LFU. Gene expression is normalized to the endogenous control gene *TFRC*. Data are expressed as median with range (*n* = 4). Values lower than *p* < 0.05 are indicated by (*) for comparisons within LFU-treated groups (different concentrations vs. LFU-only control).

**Figure 5:**
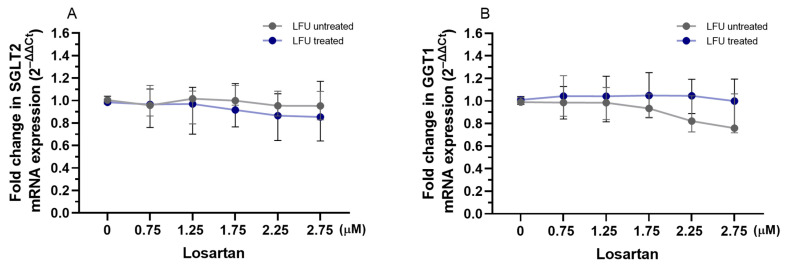
(**A**,**B**) Changes in the expression of *SGLT2* and *GGT1* genes in RPTEC/TERT1 treated with therapeutic concentrations of losartan (0.75–2.75 μM) in combination with LFU. Gene expression changes are presented as fold changes compared to losartan untreated control cells (0 μM) exposed to LFU. Gene expression is normalized to the endogenous control gene *TFRC*. Data are expressed as median with range (*n* = 4).

**Figure 6:**
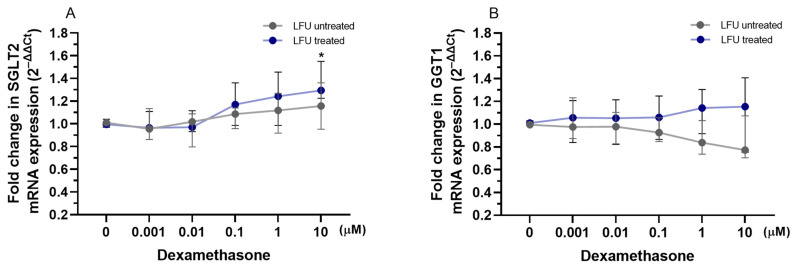
(**A**,**B**) Changes in the expression of *SGLT2* and *GGT1* genes in RPTEC/TERT1 treated with dexamethasone (0.001–10 μM) in combination with LFU. Gene expression changes are presented as fold changes compared to dexamethasone untreated control cells (0 μM) exposed to LFU. Gene expression is normalized to the endogenous control gene *TFRC*. Data are expressed as median with range (*n* = 4). Values lower than *p* < 0.05 are indicated by (*) for comparisons within LFU-treated groups (different concentrations vs. LFU-only control).

To evaluate the impact of captopril, losartan, and dexamethasone combined with LFU on the transcription of *SGLT2* and *GGT1* genes related to proximal tubule function, the mRNA expression levels in RPTEC/TERT1 cells were analyzed. The LFU treatment alone did not impact the expression of *SGLT2* and *GGT1* in RPTEC/TERT1 cells treated with the compounds (Figure 4, Figure 5 and Figure 6). Only the combination of 1.75 and 2.25 μM captopril with LFU downregulated the expression of GGT1 compared to the control group exposed to LFU (Figure 4B). Neither exposure to losartan alone nor in combination with LFU affected *SGLT2* and *GGT1* mRNA levels in RPTEC/TERT1 cells (Figure 5A,B). Nonetheless, treatment with 10 μM dexamethasone and LFU increased *SGLT2* mRNA levels compared to the control group (Figure 6A).

## 3. Discussion

The study utilized cell cultures to establish a controlled and reproducible environment for investigating specific cellular responses typically observed in vivo. HUVECs and RPTEC/TERT1 cells were utilized to gain insights into the mechanisms by which LFU enhances the therapeutic effects of losartan, captopril, and dexamethasone.

Unlike in vivo models, cell cultures eliminate the complexity of systemic interactions, enabling a precise evaluation of the mechanisms by which LFU affects cellular physiology. Endothelial cells are crucial in understanding the atherosclerosis process, as described in the introduction. This study selected HUVECs as a representative endothelial cell type, making them particularly suitable for investigating the mechanisms underlying vascular dysfunction. To investigate the interplay between endothelial dysfunction and renal physiology, given the well-established connection between impaired kidney function and circulatory diseases, renal proximal tubule epithelial cells (RPTEC/TERT1) were employed for this purpose. RPTEC/TERT1 cells retain key features of primary renal epithelial cells, including metabolic activity and ROS handling, which are critical in renal function [31].

Our study also examined the impact of dexamethasone, a glucocorticoid widely used for its anti-inflammatory and immunosuppressive properties. Dexamethasone primarily targets the cytoplasmic glucocorticoid receptors in cells, which translocate to the nucleus upon activation, modulating gene expression involved in inflammation, oxidative stress, and cell survival [32]. In contrast, the RAS inhibitors used in the study, captopril (an ACE inhibitor) and losartan (an ARB), primarily target components of the RAS located on the cell membrane (e.g., ACE and Ang II receptors). By including dexamethasone in parallel with RAS inhibitors, this study offers insight into how distinct cellular compartments—nuclear signaling pathways versus membrane-bound RAS components—respond to LFU exposure.

Previous studies have shown that LFU can influence cellular permeability and metabolic activity, enhancing drug delivery and cellular responses [33]. Ultrasound has been demonstrated to improve the cellular uptake of both low- and high-molecular-mass molecules [34]. Using sonication of the low-intensity and low-frequency (0.2–1 W/cm^2^, 69 kHz), ultrasound may modulate the vascular effects of contraction and relaxation. Ultrasound effects can be divided into thermal, chemical, and mechanical effects. While the thermal effects of ultrasound have been known for decades, recent studies have increasingly focused on its non-thermal effects due to their potential role in vascular modulation and therapeutic applications [30]. Therefore, combining LFU with drugs may synergistically modulate NO and ROS production or pathways involved in inflammation, offering insights into novel strategies to improve endothelial function and mitigate vascular dysfunction [35,36]. Our study demonstrated distinct HUVEC and RPTEC/TERT1 cell metabolic activity responses to LFU combined with captopril, losartan, and dexamethasone treatments. In HUVECs, captopril and losartan, when paired with LFU, significantly enhanced cell metabolic viability, with losartan showing a dose-dependent effect (Figure S1). Dexamethasone, however, had no impact on HUVEC viability, either alone or with LFU. The increased cell viability may be due to the synergistic effects of the pharmacological properties of captopril and losartan and the mechanical stimulation provided by LFU. Previous studies with cell cultures have shown the pro-survival effects of captopril in the upregulation of proteins (uPA, uPAR, PAI-1, and mortalin-2) involved in cell survival and immortalization [37]. Moreover, endothelial cell apoptosis may be attenuated by captopril via p38 MAP kinase inhibition [38]. In Watanabe et al.’s study, losartan was shown to suppress tumor necrosis factor-induced (TNFα) endothelial cell apoptosis by activating the VEGFR2/PI3K/Akt pathway, which may lead to improved endothelial cell survival [39]. In our study, only losartan combined with LFU improved RPTEC/TERT1 cell viability, while captopril showed no effect on these cells (Figure S2). By blocking AT_1_R, losartan prevents the downstream activation of pro-inflammatory and pro-fibrotic signaling pathways, which can otherwise contribute to cell damage and cell death in endothelial cells [40]. Interestingly, dexamethasone combined with LFU reduced RPTEC/TERT1 viability, particularly at higher concentrations (Figure S2). A decrease in viability with dexamethasone and LFU may result from a combined effect that amplifies the cytotoxicity associated with glucocorticoid exposure, especially in non-inflammatory or stress-sensitive cells like RPTEC/TERT1. Impact on viability also varies with cell type, concentration, and exposure duration. Studies on renal cell carcinoma (RCC) cell lines (NC65, ACHN, CAKI-1, CCF-RC1) indicate that dexamethasone can inhibit cell proliferation in a dose-dependent manner, through the suppression of *interleukin-6* (*IL-6*) gene expression [41]. In another study with human kidney proximal tubular cells (HK-2), dexamethasone stimulated the expression of glucocorticoid-responsive genes, such as β2-adrenoreceptors and angiotensinogen, that may impact cell function and viability [42]. In renal epithelial cells, dexamethasone does not effectively inhibit the activity of NF-κB, a key regulator of inflammation, even when the glucocorticoid receptor is active, potentially contributing to decreased cell viability [42].

Furthermore, this study demonstrated the differential effects of the drugs combined with LFU on wound healing in both cell lines. In HUVECs, captopril (1.25–2.25 μM) and higher concentrations of losartan (2.25–2.75 μM) with LFU enhanced wound closure (Figure S3). Differences in wound closure in the treated HUVECs depended on the usage of LFU. Information regarding the effects of RAS inhibitors on wound healing is limited. RAS inhibitors may positively influence wound healing processes by pleiotropic activation of endothelial cell functions and by antagonizing the ACE/Ang II/AT_1_R axis [43]. In animal models, LFU has been demonstrated to be effective in wound healing by increasing the expression of vascular growth factor expression (VEGF) and transforming growth factor (TGF)-β1 [44]. In our study, captopril with LFU showed no significant overall effect on RPTEC/TERT1 cells but demonstrated concentration-dependent variations, whereas losartan (1.75–2.75 μM) promoted wound healing (Figure S4). Interestingly, dexamethasone with LFU enhanced wound healing at lower concentrations (0.1 and 1 μM) in RPTEC/TERT1 cells, highlighting a context-dependent response (Figure S4). The septic mice model showed similar dose-dependent results. Low-dose dexamethasone had a positive effect on wound healing in septic but not healthy mice [45]. The wound healing mechanism of dexamethasone in animal models was shown to happen through decreased levels of IL-1β and TNFα, and the vasculogenesis of mature vessels was shown to improve [46].

Redox signaling molecules were evaluated, including NO secreted by HUVECs and ROS generated in RPTEC/TERT1 cells, after combined drug and LFU treatment in cell cultures. As shown in Figure S5, captopril with LFU increased NO production in HUVECs, showing a dose-dependent effect across all tested captopril concentrations. Losartan enhanced NO production only at 2.25 and 2.75 μM concentrations, while LFU alone did not influence NO synthesis in losartan-treated cells. Momose et al. demonstrated that captopril enhanced the production of NO in endothelial cells from human thoracic aortas [47]. Research with animal models has shown that some AT_1_ receptor blockers (ARBs) could upregulate endothelial NO synthase (eNOS), reduce eNOS uncoupling, and restore BH_4_, an essential cofactor to generate NO bioavailability [48,49]. The combination of RAS inhibitors and LFU may work synergistically to enhance NO production, as we demonstrated with the obtained results with the combination of captopril and LFU. In the study by Altland et al., mechanical stimulation from LFU has been shown to increase eNOS activity in endothelial cells, resulting in subsequent elevated NO synthesis [26]. Additionally, our study assessed ROS production in RPTEC/TERT1 cells in response to the treatment. The findings suggest that LFU modulates ROS production differently depending on the compound used, with a suppressive effect observed for RAS inhibitors and a stimulatory effect for dexamethasone, highlighting its role in context-specific oxidative stress regulation. LFU significantly reduced ROS levels in RPTEC/TERT1 cells treated with captopril at concentrations of 1.75 and 2.25 μM, indicating a potential antioxidative effect (Figure S6). Captopril’s beneficial effects against ROS have been demonstrated in a few studies, highlighting its effect on NADPH oxidase activity or reduction of p22phox, a subunit of NADPH oxidase [50,51]. However, high-intensity LFU may promote the generation of cavitation bubbles, increase cavitation efficiency, and subsequently amplify ROS production, thus modifying ROS levels when combined with captopril [52]. Similarly, LFU decreased ROS production in cells treated with 1.75–2.75 μM concentrations of losartan, while LFU alone did not affect ROS production (Figure S6). Pialoux et al. demonstrated that losartan-mediated AT_1_ receptor blockade effectively prevents the rise in oxidative stress caused by intermittent hypoxia [53]. Another study with cell cultures affirmed a notable decrease in ROS production following losartan treatment [54]. Conversely, combining dexamethasone with LFU led to increased ROS production across 0.1–10 μM concentrations, with variability observed at 0.001 and 0.01 μM concentrations (Figure S6). Other studies with cell cultures have indicated dexamethasone’s impact on ROS production to be cell type-specific and context-dependent [55,56].

The expression of adhesion molecules and inflammation-related genes in HUVECs was assessed after treatment with a combination of drugs and LFU in HUVECs. The investigated genes are crucial for regulating endothelial cell adhesion, inflammation, and the metabolism of lipids and eicosanoids, highlighting their role in cardiovascular and metabolic health. When combined with LFU, captopril decreased the expression of *VCAM-1*, *ICAM-1*, and *PTGS2* in a dose-dependent manner, suggesting a potential anti-inflammatory effect (Figure 1). LFU treatment alone was shown to alter the expression of these genes. The mentioned adhesion molecules are crucial in the development of atherosclerosis by promoting monocyte adhesion to endothelial cells, triggering inflammation, and contributing to plaque formation and vascular dysfunction [57]. PTGS2 plays a key role in prostaglandin synthesis and mediating vasodilation, all of which are critical for the inflammatory response. Additionally, captopril and LFU increased *CYP4F2* expression, which is involved in the resolution of inflammation and metabolism of eicosanoids [58]. Captopril was also shown to have anti-inflammatory properties in hypertensive rat models [46,50]. On the other hand, as presented in Figure 2, losartan with LFU exhibited a more selective effect. While losartan and LFU did not affect *ICAM-1* and *PTGS1* expression, a 2.75 μM concentration of losartan reduced *VCAM-1* expression. Notably, losartan combined with LFU also reduced *PTGS2* expression in a dose-dependent manner, similar to captopril. However, losartan’s impact on *CYP4F2* expression differed. Variability on *CYP4F2* expression depended on the LFU application, with only 1.75–2.75 μM concentrations reducing *CYP4F2* expression compared to control. These findings indicate that RAS inhibitors have an effect on inflammatory-related genes; however, their effects on endothelial cells differ in specificity and magnitude under LFU treatment. Dexamethasone, when combined with LFU, exhibited a distinct pattern of gene regulation compared to captopril and losartan (Figure 3). Dexamethasone with LFU downregulated *VCAM-1* and *ICAM-1* expression exclusively at higher concentrations. Mullins et al. similarly showed 1 μM concentration of dexamethasone’s effectiveness in reducing adhesion molecule expression in endothelial cells [59]. An anti-inflammatory effect was observed with dexamethasone combination with LFU that reduced *PTGS2* expression with the used range of concentrations. Dexamethasone showed context-dependent and concentration-specific effects compared to the more consistent actions of captopril and losartan in modulating inflammatory gene expression under LFU treatment.

As a final point, in this study, we investigated the effects of captopril, losartan, and dexamethasone combined with LFU on genes relevant to renal proximal tubule function; the mRNA expression levels of sodium-glucose transport protein 2 (SGLT2) and gamma-glutamyltransferase 1 (GGT1) were analyzed in RPTEC/TERT1 cells. These genes are critical for renal glucose reabsorption from the renal filtrate back into the bloodstream and glutathione metabolism, which is essential for cellular antioxidant defense, respectively [60,61]. Modulation of *SGLT2* expression by pharmacological agents has implications for renal glucose handling, potentially influencing diabetic nephropathy progression [62]. LFU alone did not alter the expression of both genes. However, 1.75 and 2.25 μM captopril combined with LFU reduced *GGT1* expression (Figure 4). Although there is no direct link between captopril and GGT1, the effect is most likely due to anti-inflammatory effects and inhibited formation of Ang II that may impact GGT1 transcription [63]. Dexamethasone combined with LFU at a concentration of 10 μM increased SGLT2 mRNA levels, suggesting a potential role in modulating glucose transport under these conditions (Figure 6). It was found that inhibition of SGLT2 in human diabetic nephropathy patients had a positive effect on inflammatory response (via inhibition of CD68, p65, TLR4, MCP-1, and osteopontin) [64]. The upregulation of *SGLT2* could be a compensatory mechanism driven by the glucocorticoid’s known effects on renal function, as dexamethasone has been reported to alter renal transporter activity [65].

The potential of LFU in enhancing drug delivery, which may improve targeted therapies for endothelial and renal cellular impairments, is partly supported by this study. The ability of LFUs to modulate oxidative stress and inflammation further supports its role in optimizing treatments for vascular and renal dysfunction [66]. This also opens opportunities for research and the development of innovative LFU instruments designed to address the unique needs of individual patients. LFU has been explored in clinical settings for improving drug bioavailability, suggesting its possible integration into current therapeutic protocols [67]. Current research indicates that LFU can promote vasodilation and reduce inflammation [36], potentially improving microvascular function. LFU has been observed to enhance the bioavailability of NO, which may contribute to improved outcomes in ischemic conditions. These findings suggest that LFU could be a non-invasive therapeutic modality to ameliorate endothelial function and support vascular recovery in patients with ischemic heart disease and CMD. Further optimization of ultrasound parameters and exploration of additional drug combinations could enhance its therapeutic applicability. As ACE inhibitors and ARBs are used for hypertension, heart failure, and post-MI management, particularly in patients with CMD and endothelial dysfunction, investigating their synergy with LFU could provide insights into the non-invasive enhancement of both vascular and renal function. This study only focused on short-acting ACE inhibitor captopril and ARB losartan due to their distinct pharmacological profiles and clinical relevance. With its rapid onset of action, captopril improved the evaluation of acute cellular responses to RAS inhibition. Meanwhile, losartan was selected not only for its selective blockade of AT_1_ receptors but also for its additional uricosuric properties, making it especially beneficial for patients with coexisting chronic kidney disease and hyperuricemia. Therefore, future research may focus on translating these findings into clinical applications for cardiovascular and renal diseases.

Our study is limited to in vitro models (HUVECs and RPTEC/TERT1), necessitating further in vivo validation to confirm the therapeutic potential of LFU–drug combinations. The precise molecular mechanisms underlying LFU’s effects require deeper investigation, particularly regarding signaling pathways.

## 4. Materials and Methods

### 4.1. Cell Culture Cultivation

**Figure 7:**
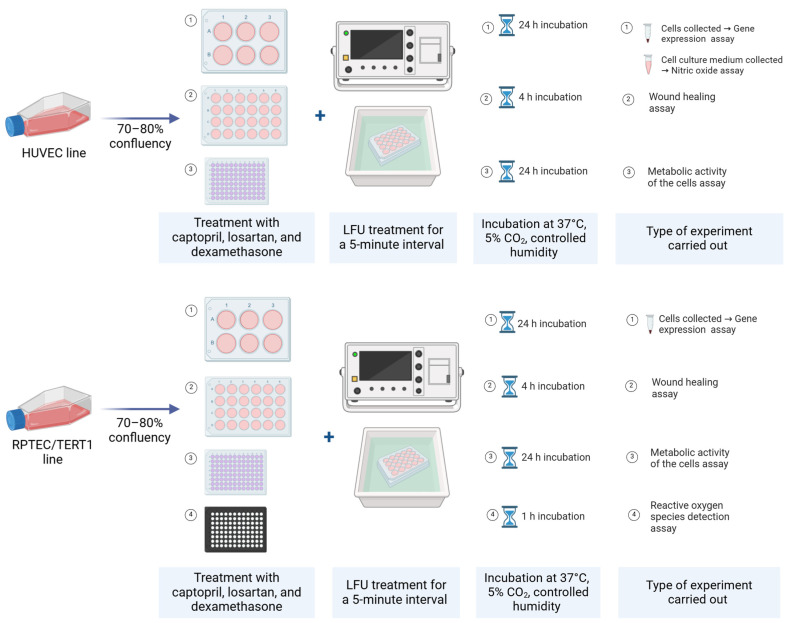
Experimental design with cell cultures.

For the study, a human umbilical vein endothelial cell (HUVEC) line sourced from Gibco (Life Technologies, Carlsbad, CA, USA) and a human renal proximal tubular epithelial cell line RPTEC/TERT1 (Evercyte, Vienna, Austria) were utilized. HUVECs were maintained in Human Large Vessel Endothelial Cell Basal Medium (HLVECBM, Gibco by Life Technologies, Carlsbad, CA, USA) supplemented with Large Vessel Endothelial Supplement (LVES) and a penicillin/streptomycin antibiotic solution (10,000 U/mL, Gibco by Life Technologies, Carlsbad, CA, USA). RPTEC/TERT1 were sustained in DMEM/F-12 basal medium (1:1 mixture) (Gibco by Life Technologies, Carlsbad, CA, USA) supplemented with 4% fetal bovine serum (FBS), 2 mM L-alanyl-L-glutamine, 1X Insulin-Transferrin-Selenium (ITS-G), 10 ng/mL human epidermal growth factor, 25 ng/mL hydrocortisone, and 100 μg/mL G418 sulfate. Once the cells reached 80% confluency in T-75 flasks, they were transferred 6-, 24-, or 96-well culture plates (Nunclon Delta Surface, Thermo Fisher Scientific, Waltham, MA, USA), depending on the type of experiment, and incubated at 37 °C with 5% CO_2_ and controlled humidity (Figure 7). Enzymatic detachment of both cell lines was performed using 0.05% trypsin-EDTA (Gibco by Life Technologies, Carlsbad, CA, USA). Cell concentration and viability were assessed with the Countess II FL automatic cell counter (Invitrogen, Waltham, MA, USA) before seeding. Cell proliferation was monitored using the EVOS XL Core imaging system (Invitrogen, Waltham, MA, USA). All experiments with HUVECs were carried out with cells at passages 3–6. RPTEC/TERT1 cells were used from passage 52 onwards. All cell culture experiments were conducted at the Institute of Cardiology of the Lithuanian University of Health Sciences.

### 4.2. Treatment of Cell Cultures with Drugs Combined with Low-Frequency Ultrasound (LFU)

A captopril stock solution (Sigma-Aldrich, St. Louis, MO, USA, Cat# C4042) was prepared by dissolving the compound in Phosphate-Buffered Saline (PBS, pH 7.4, Gibco by Life Technologies, Carlsbad, CA, USA). Losartan potassium (Sigma-Aldrich, St. Louis, MO, USA, Cat# PHR1602) was dissolved in dimethyl sulfoxide (DMSO, Sigma-Aldrich, St. Louis, MO, USA). Dexamethasone 4 mg/mL infusion solution was used for the study. Working solutions of all used compounds were prepared in a complete culture medium. HUVEC and RPTEC/TERT1 cell cultures were exposed to final concentrations of 0.25–2.25 µmol/L captopril, 0.75–2.75 µmol/L losartan, and 0.001–10 µmol/L dexamethasone in the culture medium. Afterwards, treated cells in culture dishes were placed in an incubator with an ultrasonic bath filled with distilled water, where LFU of 69 kHz with an intensity of 5–10 mW/cm^2^ was transmitted for a 5 min interval. Low-intensity US was used in order not to disrupt the structure of the cultures. Cells were further incubated under usual conditions for 24 h.

### 4.3. LFU Device Design

**Figure 8:**
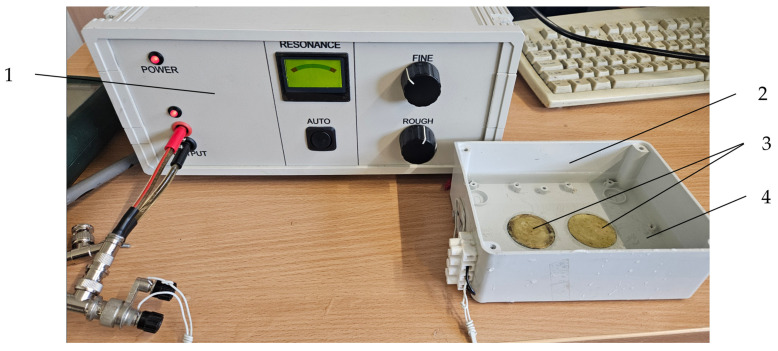
Ultrasonic bath with signal controller: 1—signal controller; 2—ultrasonic bath; 3—piezo elements; 4—distilled water.

An ultrasonic bath of the special dimensions and technical characteristics for sonification-treated cells in culture dishes was designed with two disc-shaped bimorph-type piezoelectric actuators attached to the bottom of bath (Table 1). The application of the piezoelectric bimorph actuators was based on three design concepts: (1) higher vibration amplitudes are generated by utilizing a bimorph-type actuator with a clamped edge at the perimeter to the bottom of the bath; (2) a less scattered acoustic field is generated by the radial-mode vibration of the piezoelectric bimorph actuator; (3) more energy is generated in the radial vibration mode of the higher ultrasonic frequency. To drive a piezoelectric actuator at the resonant frequency of the higher vibration mode, a special controller was developed (Figure 8).

**Figure 9:**
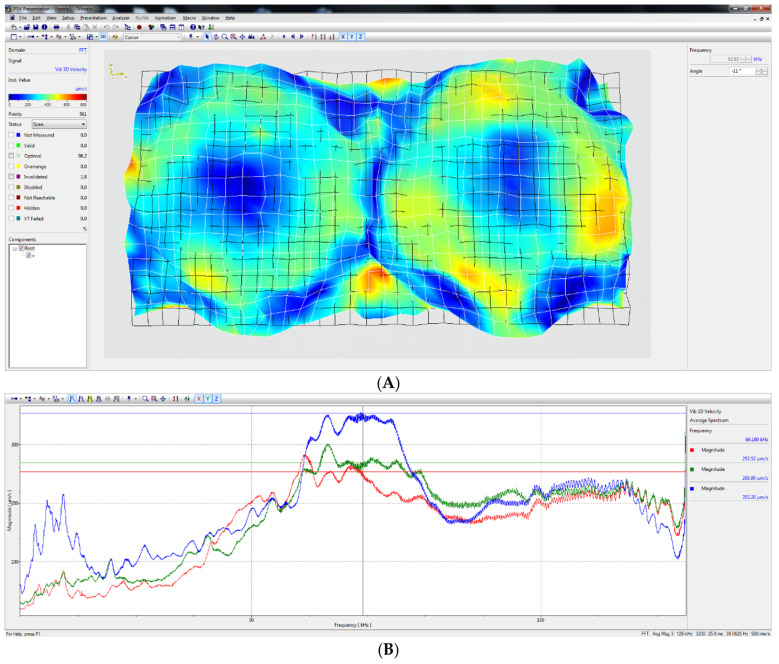
An experimental modal test was performed using a Polytec 3D scanning vibrometer. (**A**) A deflection shape of the developed transducer of the radial vibration mode at frequency 69.18 kHz. (**B**) 3D frequency response of the tested transducer, where X (red) and Y (green) are in-plain vibrations; Z (blue)—out-of-plain vibration. The blue, green and red horizontal lines show the average root mean square (RMS) values of the peak vibration in the z, x and y directions respectively, generated by the actuator in the frequency range 60–75 kHz.

**Figure 10:**
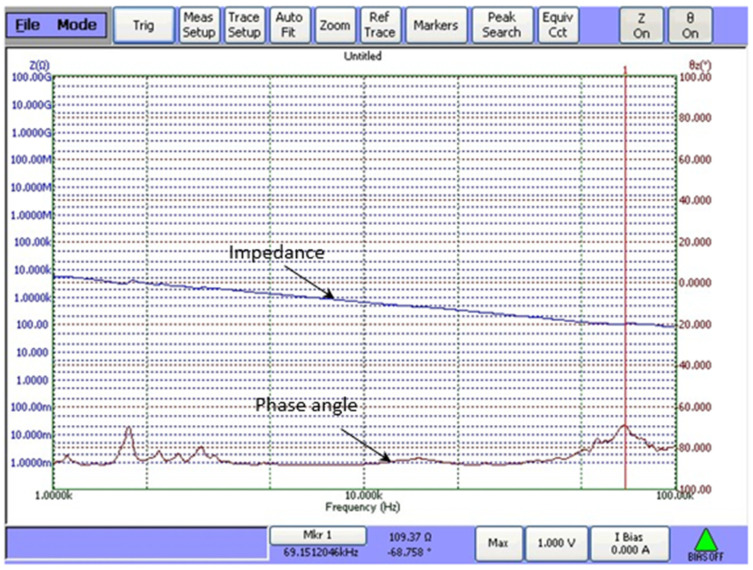
Example of electromechanical impedance of the developed transducer measuring with an impedance analyzer.

To assess the sonication process and dynamic characteristics of the developed ultrasonic bath, an experimental set-up with a Polytec 3D scanning vibrometer (Type PSV-500-3D-HV, Polytec GmbH, Waldbronn, Germany) and an impedance analyzer 6500B (Wayne Kerr Electronics Ltd., Bognor Regis, UK) were used. Measured results showed that higher amplitudes of vibration of the disc-shaped bimorph type piezoelectric actuators were generated at a resonant frequency of 69 kHz. The amplitude–frequency characteristic and electromechanical impedance of the piezoelectric actuators are shown in Figure 9 and Figure 10.

The Polytec Laser Doppler 3D scanner system (Polytec GmbH, Waldbronn, Germany) was used for the high-precision three-dimensional vibration measurement of the transducer’s radiation surface. The transducer was driven with the harmonic signal of 40 V_P-P_, and its frequency ranged from 1 kHz to 150 kHz. The amplitude–frequency characteristic of the piezoelectric transducer measured with the Polytec system is shown in Figure 9.

The electromechanical characteristics of the piezoelectric actuator were evaluated by measuring electrical impedance vs. frequency characteristics (Figure 10). This figure shows the resonant frequency at 69 kHz, which consists of the radial vibration mode measured with a Polytec system. Further, a LFU of this frequency was used for the sonification of biological tissues.

### 4.4. Metabolic Activity of the Cells

The metabolic activity of the cells was evaluated using the 3-(4,5-dimethylthiazol-2-yl)-2,5-diphenyl-2H-tetrazolium bromide (MTT) assay (Invitrogen, Waltham, MA, USA) [68]. Each experiment included triplicate wells for each drug concentration, control, and background wells. A 5 mg/mL MTT solution was added to each well containing serum-free medium, followed by a 3.5 h incubation. The resulting formazan crystals were dissolved in DMSO for 15 min on a plate shaker. Absorbance was measured spectrophotometrically at 550 nm with a reference wavelength of 620 nm using an Infinite^®^ M Plex microplate reader (Tecan, Mannedorf, Switzerland).

### 4.5. Wound-Healing Assay

HUVECs were seeded at a density of 0.4 × 10^5^ cells/cm^2^, RPTEC/TERT1—0.6 × 10^5^ cells/cm^2^ for the optical wounding experiments. After 24 h of incubation (37 °C, 5% CO_2_, humidified atmosphere), when a confluent cell layer was established, a 0.5 mm gap, mimicking a wound, was made in the cell monolayer [69]. The medium was exchanged for fresh cell culture medium without serum combined with the studied compounds. Cells were treated with LFU, as described earlier. Cell migration into the wounded areas was documented by phase contrast microscopy. Images of the wound area were taken at 0 and 4 h using the EVOS XL Core cell imaging system (Invitrogen, Waltham, MA, USA). Wound closure was quantified by measuring the area of the wound at each time point using ImageJ v1.54d software (NIH, Bethesda, MD, USA). The percentage of wound closure was calculated based on the initial wound area based on the formula:

% wound closure = [(wound area at time 0 − wound area at time x)/wound area at time 0] × 100

### 4.6. Nitric Oxide Assay

Total NO was quantitatively determined in treated HUVECs culture medium using a Nitric Oxide Assay Kit (Invitrogen, Waltham, MA, USA, Cat# EMSNO) following the manufacturer’s protocol. Quantification enabled the determination of nitrite and nitrate concentration in cell culture medium samples. The kit uses nitrate reductase to convert nitrate to nitrite, which is detected as a colored azo dye product of the Griess reaction. Absorbance was measured spectrophotometrically at 540 nm with an Infinite^®^ M Plex microplate reader. The intra-assay precision of the kit is less than 1.7% (mean of %CV), and the inter-assay precision is less than 4% for nitrite and nitrate detection, as declared by the manufacturer of this kit.

### 4.7. Reactive Oxygen Species Detection Assay

ROS production was detected in live RPTEC/TERT1 cells treated with drugs and LFU using ROS/Superoxide Detection Assay Kit (Abcam, Cambridge, UK, Cat# ab139476) according to the manufacturer’s guidelines. Cells were seeded and treated with captopril, losartan, and dexamethasone combined with LFU in 96-well clear-bottom black polystyrene microplates. Cells were exposed to drugs for 1 h, and an induction step with oxidative stress detection reagent was carried out. After 1 h of induction, fluorescence intensity was measured with Infinite^®^ M Plex microplate reader standard fluorescein (Extinction = 488 nm, Emission = 520 nm) filter set parameters at endpoint mode. Each assay included a positive control (ROS inducer pyocyanin; 25 µM), negative control (ROS inhibitor *N*-acetyl-L-cysteine; 5 mM), untreated samples (with vehicle dimethylformamide), untreated samples for autofluorescence, and background control wells. Control (untreated) samples presented only low autofluorescence signals. Cells pretreated with the ROS inhibitor (*N*-acetyl-L-cysteine) also presented low fluorescence intensity. For each RPTEC/TERT1 treatment, relative fluorescence intensity was normalized to experimental control samples.

### 4.8. Gene Expression Assay

Total RNA was isolated from cell pellets using the PureLink™ RNA Mini Kit (Invitrogen, Waltham, MA, USA) in combination with TRIzol^®^ Reagent (Invitrogen, Waltham, MA, USA), following the manufacturer’s protocol. The RNA yield and purity were assessed with a NanoDrop 2000 spectrophotometer (Thermo Fisher Scientific, Waltham, MA, USA). Genomic DNA (gDNA) was removed by treating RNA samples with deoxyribonuclease I (DNase I; Thermo Scientific, Waltham, MA, USA). Reverse transcription was performed with the High-Capacity cDNA Reverse Transcription Kit containing RNase Inhibitor (Thermo Fisher Scientific, Waltham, MA, USA), using 0.5 µg of RNA as the input, following the manufacturer’s protocol. Quantitative real-time PCR (qPCR) gene expression analysis was conducted following MIQE guidelines [70]. Reactions were set up in a 10 µL total volume, containing 5 µL of 2× Power SYBR Green PCR Master Mix (Applied Biosystems, Waltham, MA, USA), 2.5 µL of PCR-grade water, 250 nM of each primer, and 10 ng of cDNA. Primer sequences for the gene expression experiments are detailed in Table 2. The qRT-PCR program included an initial denaturation at 95 °C for 10 min, followed by 40 cycles of 95 °C for 15 s, 60.6 °C for 30 s, and 72 °C for 1 min. A melting curve was generated at the end of the reactions. All qPCR reactions were run in triplicate, and the C_T_ values were averaged. Normalized relative gene expression was calculated using the 2^−∆∆Ct^ method, with the transferrin receptor (TFRC) serving as an endogenous control for normalization. All reactions were carried out using the QuantStudio 5 real-time PCR system (Applied Biosystems, Waltham, MA, USA).

### 4.9. Statistical Analysis

Statistical analysis was conducted using GraphPad Prism V8 software (San Diego, CA, USA). Data normality was evaluated with the Shapiro–Wilk test. Since the data did not follow a normal distribution, the nonparametric Mann–Whitney U test was employed to compare differences between the two independent groups. Results are presented as a median with a range. A *p*-value of less than 0.05 was considered significant.

## 5. Conclusions

The study highlights the modulatory role of LFU in conjunction with RAS inhibitors captopril and losartan, as well as with glucocorticoid dexamethasone, on cell viability, inflammatory responses, and wound healing in vascular and renal cell line models. LFU enhanced the therapeutic effects of RAS inhibitors by promoting endothelial NO synthesis and mitigating oxidative stress. In contrast, combining LFU with dexamethasone yielded variable outcomes regarding cell survival and oxidative stress, potentially resulting in heightened cytotoxicity in renal cells. Gene expression analyses revealed distinct gene expression patterns attributable to LFU and pharmacological interventions, underscoring LFU’s anti-inflammatory potential and suggesting that LFU may augment these drugs’ pharmacokinetic or pharmacodynamic efficacy.

## Figures and Tables

**Table 1:** Characteristics of the LFU device.

Frequency	~69 kHz
Power	40 W
Capacity	0.5 L
Adjust power	40–100%
Pan size (mm)	120 × 80 × 10
Dimensions (mm)	130 × 90 × 60
Power supply	220 V/50 Hz
Timing	1–99 min
Accessories	bracket

**Table 2:** qPCR primers used for gene expression quantification.

Gene Symbol	Accession	Name	Primer	Primer Sequence
*VCAM-1*	NM_001078.4	Vascular Cell Adhesion Molecule 1	Forward	5′-AGGTGGAGATCTACTCTTTTCCT-3′
			Reverse	5′-ACACTTGACTGTGATCGGCT-3′
*ICAM-1*	NM_000201.3	Intercellular Adhesion Molecule 1	Forward	5′-GGTGCTGGTGAGGAGAGATCA-3′
			Reverse	5′-AGTCGCTGGCAGGACAAAGG-3′
*PTGS1*	NM_000962.4	Prostaglandin-Endoperoxide Synthase 1	Forward	5′-CTGGTGGATGCCTTCTCTCG-3′
			Reverse	5′-CATCTCCCGAGACTCCCTGA-3′
*PTGS2*	NM_000963.4	Prostaglandin-Endoperoxide Synthase 2	Forward	5′-CGGTGAAACTCTGGCTAGACAG-3′
			Reverse	5′-GCAAACCGTAGATGCTCAGGGA-3′
*CYP4F2*	NM_001082.5	Cytochrome P450 Family 4 Subfamily F Member 2	Forward	5′-GACAGCCATTGTCAGGAGAAACC-3′
			Reverse	5′-TGCAGGAGGATCTCATGGTGTC-3′
*SGLT2*/ *SLC5A2*	NM_003041.4	Solute Carrier Family 5 (Sodium/ Glucose Cotransporter), Member 2	Forward	5′-AGTGCCTGCTCTGGTTTTGT-3′
			Reverse	5′-TTAGGCATAGAAGCCCCAGA-3′
*GGT1*	NM_013421.3	Gamma-Glutamyltransferase 1	Forward	5′-TGCTCGAAGATTGGGAGGGATG-3′
			Reverse	5′-ACACAACAGGGCTGCAATGG-3′
*TFRC*	NM_003234.4	Transferrin Receptor	Forward	5′-ACTTGCCCAGATGTTCTCAGAT-3′
			Reverse	5′-CGAAAGGTATCCCTCTAGCCAT-3′

## Data Availability

The original contributions presented in this study are included in the article/Supplementary Materials. Further inquiries can be directed to the corresponding author.

## Supplementary Materials

The following supporting information can be downloaded at: https://www.mdpi.com/article/10.3390/ph18030404/s1, Figure S1. Effect of captopril, losartan, and dexamethasone with LFU on HUVECs viability after 24 h using MTT assays. (A) HUVECs viability after treatment with captopril (0.25–2.25 μM) and LFU. (B) HUVECs viability after treatment with losartan (0.75–2.75 μM) and LFU. (C) HUVECs viability after treatment with dexamethasone (0.001–10 μM) and LFU; Figure S2. Effect of captopril, losartan, and dexamethasone with LFU on RPTEC/TERT1 cell viability after 24 h using MTT assays. (A) RPTEC/TERT1 cell viability after treatment with captopril (0.25–2.25 μM) and LFU. (B) RPTEC/TERT1 cell viability after treatment with losartan (0.75–2.75 μM) and LFU. (C) RPTEC/TERT1 cell viability after treatment with dexamethasone (0.001–10 μM) and LFU; Figure S3. Effect of captopril, losartan, and dexamethasone with LFU on HUVECs wound healing captured 4 h after wounding. (A) HUVECs wound closure after treatment with captopril (0.25–2.25 μM) and LFU. (B) HUVECs wound closure after treatment with losartan (0.75–2.75 μM) and LFU. (C) HUVECs wound closure after treatment with dexamethasone (0.001–10 μM) and LFU; Figure S4. Effect of captopril, losartan, and dexamethasone with LFU on RPTEC/TERT1 wound healing captured 4 h after wounding. (A) RPTEC/TERT1 cell wound closure after treatment with captopril (0.25–2.25 μM) and LFU. (B) RPTEC/TERT1 cell wound closure after treatment with losartan (0.75–2.75 μM) and LFU. (C) RPTEC/TERT1 cell wound closure after treatment with dexamethasone (0.001–10 μM) and LFU; Figure S5. Effect of captopril, losartan, and dexamethasone with LFU on HUVECs NO production. (A) HUVECs NO production after treatment with captopril (0.25–2.25 μM) and LFU. (B) HUVECs NO production after treatment with losartan (0.75–2.75 μM) and LFU. (C) HUVECs NO production after treatment with dexamethasone (0.001–10 μM) and LFU; Figure S6. Effect of captopril, losartan, and dexamethasone with LFU on RPTEC/TERT1 cell ROS production. (A) RPTEC/TERT1 cell ROS production after treatment with captopril (0.25–2.25 μM) and LFU. (B) RPTEC/TERT1 cell ROS production after treatment with losartan (0.75–2.75 μM) and LFU. (C) RPTEC/TERT1 cell ROS production after treatment with dexamethasone (0.001–10 μM) and LFU.
