# Supplementary material for: Synergistic Effects of Low-Frequency Ultrasound and Therapeutic Agents on Endothelial and Renal Cells: Emphasis on Cell Functionality, Oxidative Stress, and Inflammatory Markers
Source: Pharmaceuticals (Basel). 2025 Mar 13;18(3):404. doi: 10.3390/ph18030404 (PMC11945135; doi:10.3390/ph18030404)
Supplement: Supplementary file 1 [file pharmaceuticals-18-00404-s001.zip › pharmaceuticals-3500924-supplementary.pdf]

Supplementary Materials

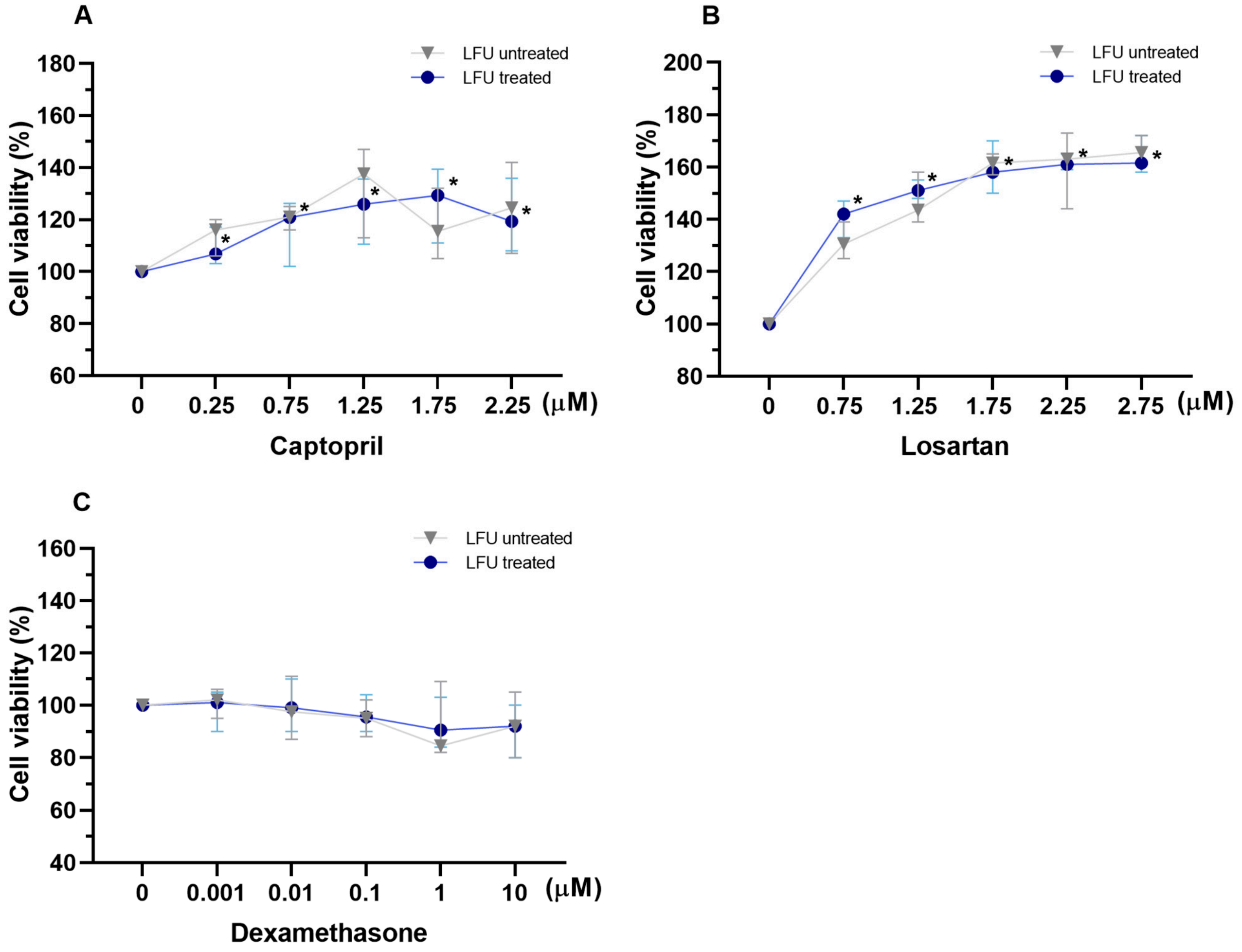

Figure S1. Effect of captopril, losartan, and dexamethasone with LFU on HUVECs viability after 24 h using MTT assays. **(A)** HUVECs viability after treatment with captopril (0.25–2.25 μM) and LFU. **(B)** HUVECs viability after treatment with losartan (0.75–2.75 μM) and LFU. **(C)** HUVECs viability after treatment with dexamethasone (0.001–10 μM) and LFU. Data are expressed as median with range ($n = 4$). Values lower than $p<0.05$ are indicated by (*) for comparisons within LFU-treated groups (different concentrations *vs.* LFU-only control).

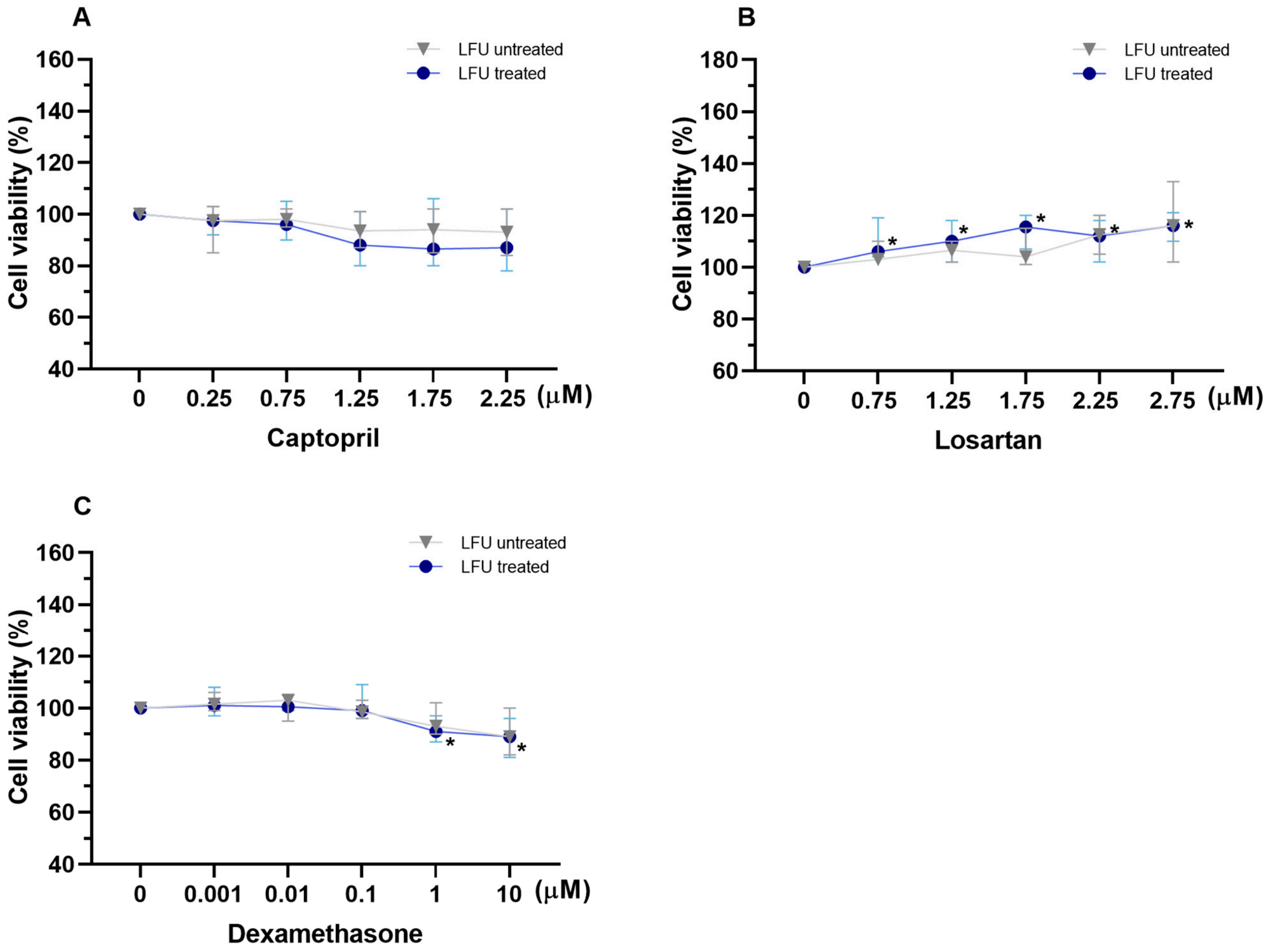

Figure S2. Effect of captopril, losartan, and dexamethasone with LFU on RPTEC/TERT1 cell viability after 24 h using MTT assays. **(A)** RPTEC/TERT1 cell viability after treatment with captopril (0.25–2.25 μM) and LFU. **(B)** RPTEC/TERT1 cell viability after treatment with losartan (0.75–2.75 μM) and LFU. **(C)** RPTEC/TERT1 cell viability after treatment with dexamethasone (0.001–10 μM) and LFU. Data are expressed as median with range ($n = 4$). Values lower than $p<0.05$ are indicated by (*) for comparisons within LFU-treated groups (different concentrations *vs.* LFU-only control).

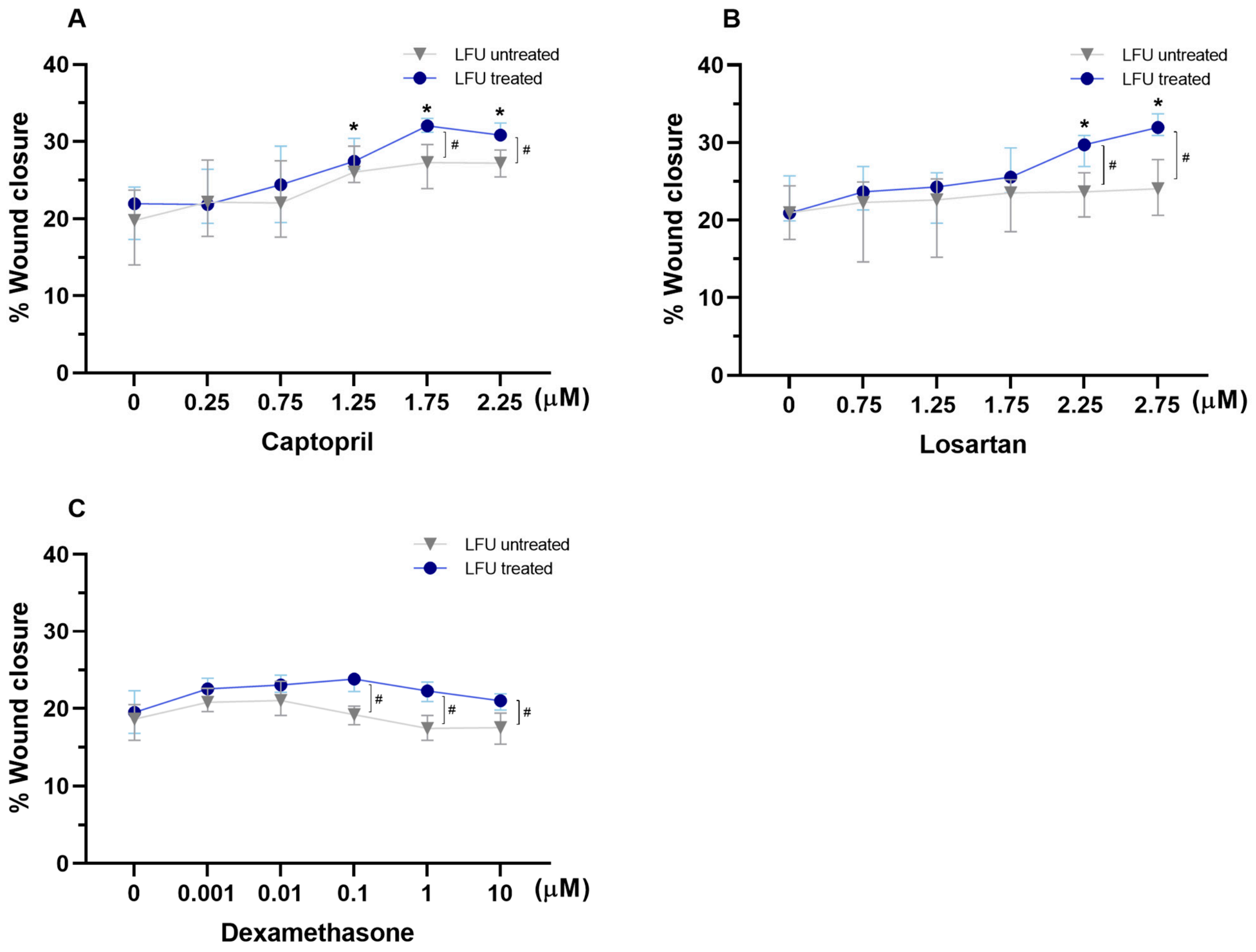

Figure S3. Effect of captopril, losartan, and dexamethasone with LFU on HUVECs wound healing captured 4 h after wounding. **(A)** HUVECs wound closure after treatment with captopril (0.25–2.25 μM) and LFU. **(B)** HUVECs wound closure after treatment with losartan (0.75–2.75 μM) and LFU. **(C)** HUVECs wound closure after treatment with dexamethasone (0.001–10 μM) and LFU. Data are expressed as median with range ($n = 4$). Values lower than $p<0.05$ are indicated by (*) for comparisons within LFU-treated groups (different concentrations *vs.* LFU-only control) and by (#) for comparisons between LFU-treated and LFU-untreated groups.

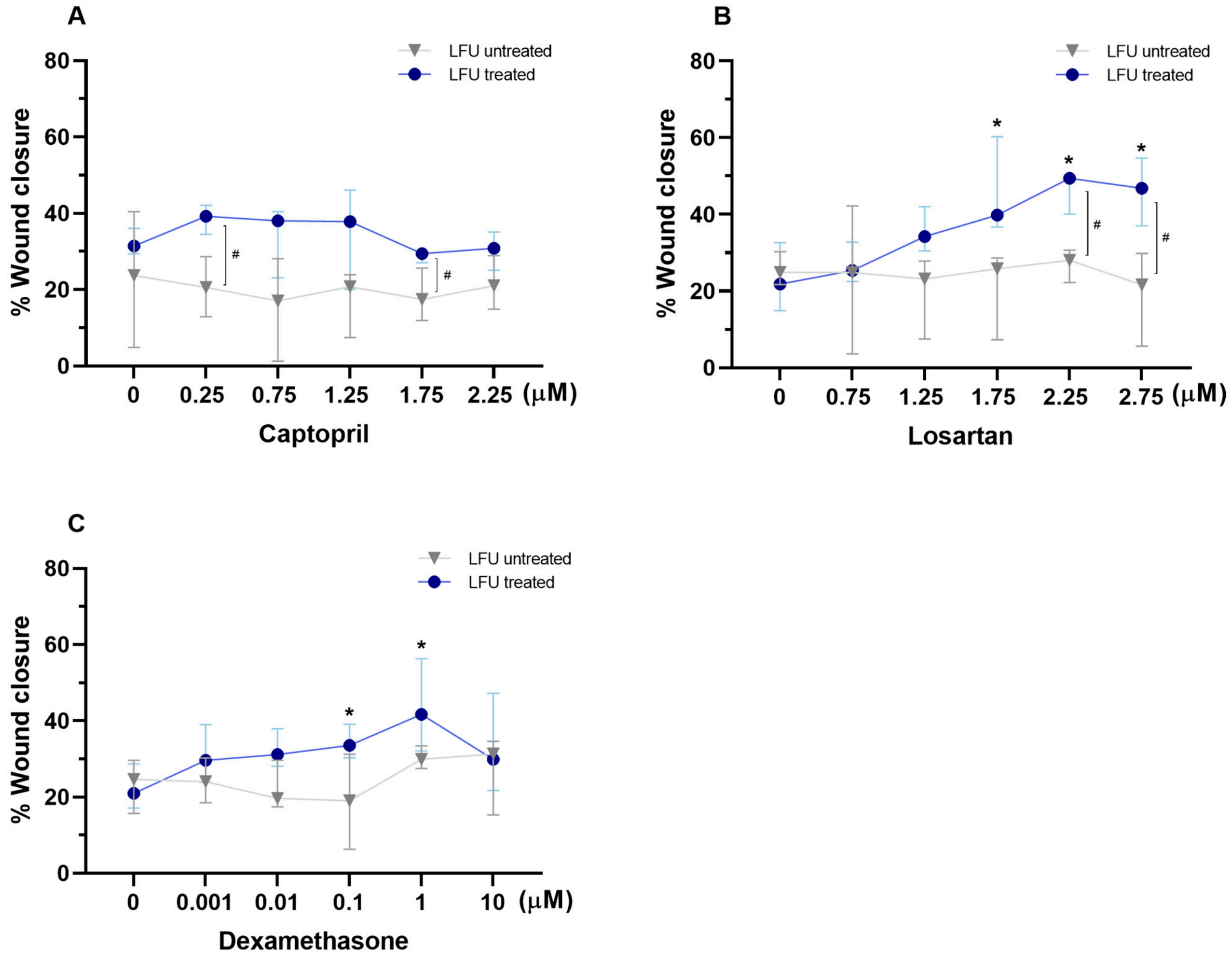

Figure S4. Effect of captopril, losartan, and dexamethasone with LFU on RPTEC/TERT1 wound healing captured 4 h after wounding. **(A)** RPTEC/TERT1 cell wound closure after treatment with captopril (0.25–2.25 μM) and LFU. **(B)** RPTEC/TERT1 cell wound closure after treatment with losartan (0.75–2.75 μM) and LFU. **(C)** RPTEC/TERT1 cell wound closure after treatment with dexamethasone (0.001–10 μM) and LFU. Data are expressed as median with range ($n = 4$). Values lower than $p<0.05$ are indicated by (*) for comparisons within LFU-treated groups (different concentrations *vs.* LFU-only control) and by (#) for comparisons between LFU-treated and LFU-untreated groups.

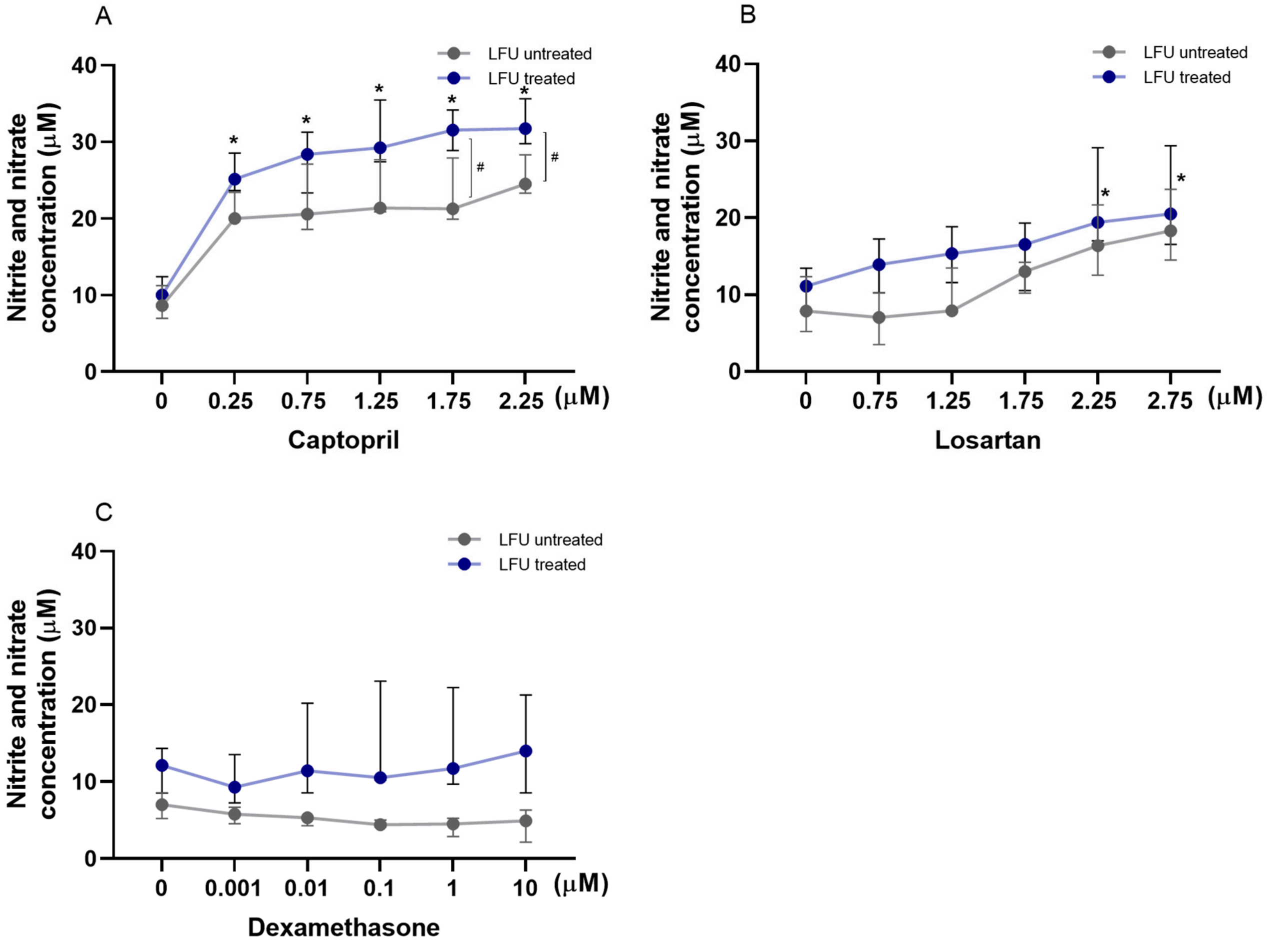

Figure S5. Effect of captopril, losartan, and dexamethasone with LFU on HUVECs NO production. **(A)** HUVECs NO production after treatment with captopril (0.25–2.25 μM) and LFU. **(B)** HUVECs NO production after treatment with losartan (0.75–2.75 μM) and LFU. **(C)** HUVECs NO production after treatment with dexamethasone (0.001–10 μM) and LFU. Data are expressed as median with range ($n = 4$). Values lower than $p<0.05$ are indicated by (*) for comparisons within LFU-treated groups (different concentrations *vs.* LFU-only control) and by (#) for comparisons between LFU-treated and LFU-untreated groups.

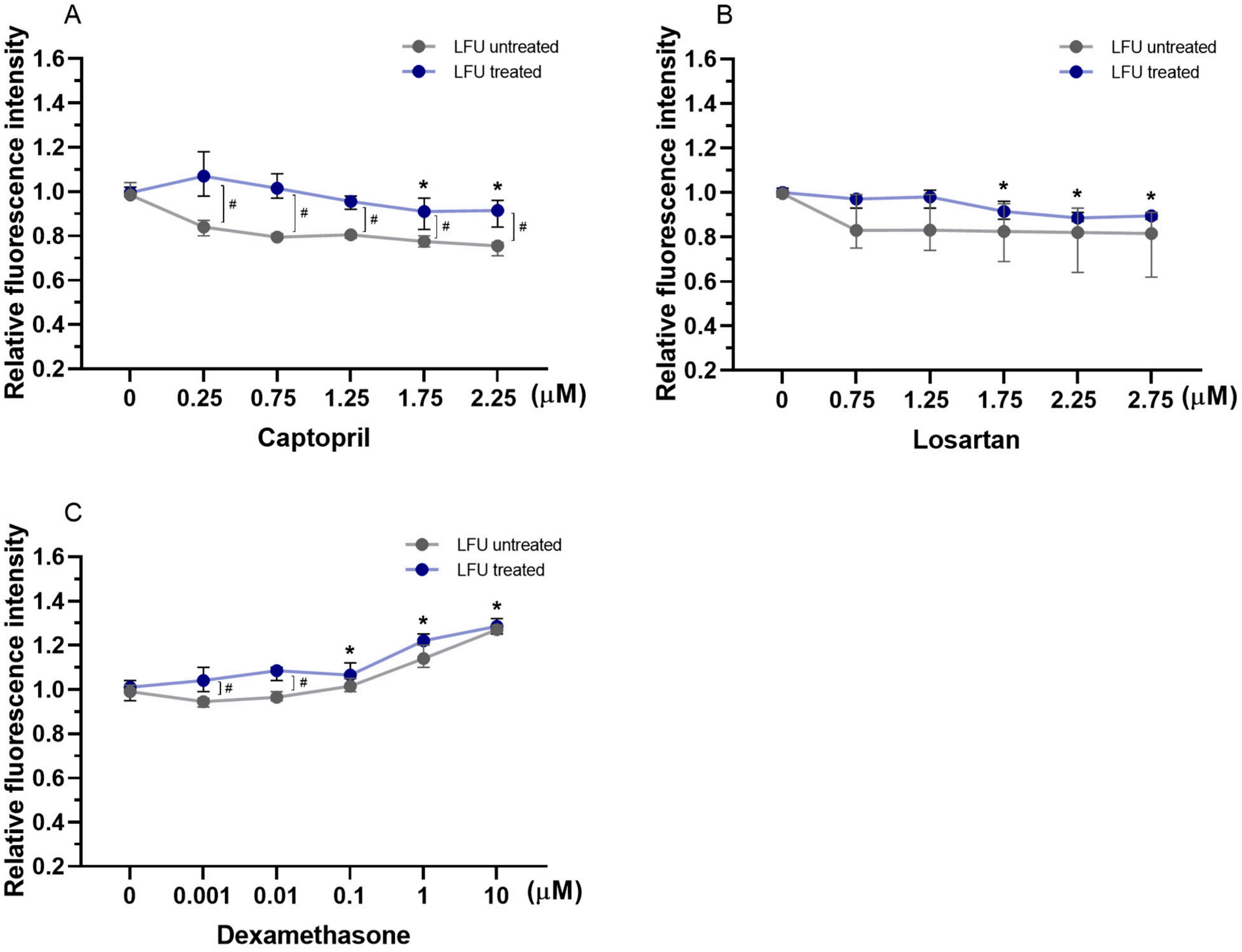

Figure S6. Effect of captopril, losartan, and dexamethasone with LFU on RPTEC/TERT1 cell ROS production. **(A)** RPTEC/TERT1 cell ROS production after treatment with captopril (0.25–2.25 μM) and LFU. **(B)** RPTEC/TERT1 cell ROS production after treatment with losartan (0.75–2.75 μM) and LFU. **(C)** RPTEC/TERT1 cell ROS production after treatment with dexamethasone (0.001–10 μM) and LFU. Data are expressed as median with range ($n = 4$). Values lower than $p<0.05$ are indicated by (*) for comparisons within LFU-treated groups (different concentrations *vs.* LFU-only control) and by (#) for comparisons between LFU-treated and LFU-untreated groups.
